# Experiences, coping strategies and perspectives of people in Malaysia during the COVID-19 pandemic

**DOI:** 10.1186/s12889-023-15892-5

**Published:** 2023-06-06

**Authors:** Phaik Kin Cheah, Mohamed Bella Jalloh, Phee-Kheng Cheah, Darlene Ongkili, Mira Leonie Schneiders, Anne Osterrieder, Pimnara Peerawaranun, Naomi Waithira, Alun Davies, Mavuto Mukaka, Phaik Yeong Cheah

**Affiliations:** 1 Faculty of Arts & Social Science, Universiti Tunku Abdul Rahman, Jalan Universiti, Bandar Barat, 31900 Kampar, Perak, Malaysia; 2 Mahidol Oxford Tropical Medicine Research Unit, Faculty of Tropical Medicine, Mahidol University, Bangkok, 10400 Thailand; 3 Centre for Tropical Medicine & Global Health, Nuffield Department of Medicine, University of Oxford, Oxford, UK; 4 Sabah Women & Children’s Hospital, Ministry of Health, Kota Kinabalu, Sabah, Malaysia; 5 Loh Guan Lye Specialists Centre, Penang, Malaysia; 6 Queen Elizabeth Hospital, Ministry of Health, Kota Kinabalu, Sabah, Malaysia; 7 The SoNAR-Global Network, Mahidol University, 10400 Bangkok, Thailand; 8 Socio-Ecological Health Research Unit, Department of Public Health, Institute of Tropical Medicine, Antwerp, Belgium

**Keywords:** COVID-19, Lockdown, Malaysia, Pandemic, Public health measures, Vaccine

## Abstract

**Background:**

A nationwide Movement Control Order (MCO) was enforced in Malaysia on 18 March 2020 in view of the global COVID-19 pandemic. Malaysia implemented various public health measures and later raced against time to administer COVID-19 vaccines when they became available. As a result of various public health measures to curb the spread of the virus, people in Malaysia faced unprecedented circumstances and new challenges. This study addressed the knowledge gap in our understanding the experiences, coping strategies and perspectives of the people in Malaysia about infection countermeasures by investigating their experiences during the COVID-19 pandemic.

**Methods:**

A sequential mixed method approach was used to conduct an online survey and in-depth interviews among residents in Malaysia. A total of 827 respondents participated in the online survey from 1st May to 30th June 2020. Nineteen in-depth interviews were conducted online and by phone with key informants and members of the public, who were selected through maximum variation purposive sampling between 2nd May 2020 to 20th December 2021. The semi-structured interviews employed a phenomenological approach and transcripts were analysed using thematic analysis. The survey data were analysed using descriptive statistics in Stata 15.0.

**Results:**

The survey reflected significant economic impacts of the pandemic, the maximum number of days that people could cope during the MCO, and their coping strategies, which generally entailed changes in lifestyle. The internet and social media were vital platforms to mitigate against the impact of public health measures. Thematic analysis of the interview data revealed participant experiences and perceptions of COVID-19 and public health measures in four main themes: (1) work and business; (2) emotional impact (3) coping with change and (4) the COVID-19 vaccine.

**Conclusions:**

This study provides insights into the experiences, coping strategies and perspectives of people in Malaysia living through the first-ever MCO during the COVID-19 pandemic. Such insights into COVID-19-related public health measures are pertinent for successfully planning and implementing future responses to pandemics.

**Supplementary Information:**

The online version contains supplementary material available at 10.1186/s12889-023-15892-5.

## Background

Malaysia is an upper middle-income country with a population of 32 million people. The population is made up of people of various ethnicities such as Malays, Chinese and Indians. A significant gap exists on the perspectives of the people in Malaysia during the COVID-19 pandemic. A nationwide Movement Control Order (MCO) was enforced under the country’s Prevention and Control of Infectious Diseases Act 1988 on 18th March 2020. The MCO required people to adhere to safety measures such as social distancing, wearing masks, contact tracing, and limiting in-country and international travel. The Malaysian government has attempted to administer COVID-19 vaccine and booster shots as rapidly as possible since receiving the first shipment of vaccines in February 2021.

In view of the global COVID-19 pandemic, the Malaysian government also implemented several biomedical and safety measures. The first MCO to restrict the spread of the virus, implemented between 18 March and 28 April 2020 [1] included restrictions on mass gatherings such as sports and other social events, the temporary closure of worship houses, educational institutions and international borders to foreign tourists, COVID-19 health screenings, and self-quarantine for travellers returning home from abroad. The MCO also meant that only one household member could leave their home to purchase essential supplies [2]. The MCO had two primary goals. First, to slow the virus’s spread and enable health officials to track, trace, and isolate COVID-19 patients. Second, to maximise screening of targeted clusters to prevent the virus from spreading further, all within the virus’s incubation period [1].

As the case count continued to rise exponentially in April 2020 due to widespread community transmission, the Malaysian government instituted an Enhanced Movement Controlled Order (EMCO) for specific areas designated as hotspots, including Kluang, Hulu Langat, Menara City One, Selangor Mansion, and Malayan Mansion in Kuala Lumpur [2]. Communities under EMCO were placed under lockdown, and people were not permitted to leave their homes or receive visitors. The authorities provided financial stimulus packages and food items to assist families in these areas [2].

Later, with the emerging of new variants [3] the government increased surveillance and testing, including gene sequencing to detect emerging COVID-19 variants in July 2021. Virtual COVID-19 assessment centres were established to monitor mild and asymptomatic patients who required self-isolation. Public-private partnerships were also utilized by the Ministry of Health, empowering private physicians to help manage mild cases and allowing private laboratories to conduct screenings [4]. This, combined with the aggressive clinical management of COVID-19 cases in hospitals, utilising stringent infection, prevention, and control measures, as well as uptake of internationally recognised guidelines for treating symptomatic infections with a combination of effective drugs such as dexamethasone, resulted in a rapid improvement in recovery rates [4]. Malaysia reported a 0.8% case fatality rate as of 18 July 2021 but it has declined to 0.2% in October 2022 [4].

In February 2021, widespread mass vaccinations commenced based on priority groups. In phase one, the top priority group receiving vaccinations included frontline public and private healthcare workers, followed by the second priority group, such as frontline staff providing essential services, as well as defence and security personnel [5]. In phase two, first, the remainder of healthcare workers, essential service workers, and defence and security personnel were vaccinated, followed by those aged 60 years and above, individuals with chronic conditions (such as heart disease, obesity, diabetes, hypertension), as well as individuals with disabilities [5]. Phase three was opened to all adults aged 18 years and over, including non-citizens, prioritising residents of high-transmission areas [5].

In addition to its direct and indirect effects on healthcare systems, COVID-19 and associated public health measures have had a wide range of social and economic consequences, especially in low- and middle-income countries [6]. First, drastic public health measures disrupted businesses, resulting in job losses or reduced work hours, lowering household income and pushing people into poverty [6]. Second, enacted public health policies disproportionately affected vulnerable groups such as the poorest people, older people, people with chronic health conditions, and slum settlers [6, 7]. Lastly, informal workers shouldered a disproportionate share of the burden due to their lack of social security coverage [7]. They are among the most vulnerable groups during and after the crisis, as they lack the financial resources necessary to buffer against income shocks [7].

One complexity of the COVID-19 pandemic also facing Malaysia revolved around balancing the health benefits and economic costs associated with controlling the virus’s spread through safety measures [8, 9]. Thus, the implementation of safety measures must be balanced against their socio-economic implications at societal and individual levels, particularly in contexts with a high reliance on informal sectors, such as in Malaysia. Consideration thus needs to be given to the effects on physical health, the economy, psychosocial well-being, human rights, food security, socioeconomic disparities, and the treatment of other health conditions besides COVID-19. The projected socio-economic consequences of COVID-19 in Malaysia included, for instance, a decline in aviation and tourism related economic activities; a fall in foreign trade; a fall in stock market investment; a fall in consumer spending and cash flow problems due to the MCO enactment, leading to layoffs and further reductions of domestic demand [10]. The MCO also adversely affected immigrants and refugees, most of whom work in the informal sector without job protection or access to government assistance, as many lost their income due to temporary business closures [11].

Even in high-income countries with readily available health and economic data, maintaining the economy while mitigating the spread of the virus has proven hugely challenging. Conversely, low- and middle-income countries, which are typically resource-limited lack accurate and consistent data on the spread of COVID-19 as well as the health and economic effects of COVID-19 public health measures [6].

While COVID-19 has created major threats on health and livelihoods across the globe, the unique disruptive forces of the pandemic have also stimulated emerging opportunities for novel solutions during worldwide crisis and post-pandemic development [12, 13]. At large, the pandemic has leveraged civil society and private innovation by building resilience of vulnerable populations through social protection initiatives providing them medical and financial support [13].

Despite the rapidly evolving evidence regarding COVID-19 health consequences and macroeconomic implications, little has been researched and written on the social and economic consequences of COVID-19 public health measures for individuals and communities, particularly in Malaysia [6]. To this end, developing an understanding of contextual factors affecting people’s livelihoods is critical for informing social schemes and policies during this ongoing pandemic. Hence, the purpose of this study was to investigate experiences, coping strategies and perspectives of the people in Malaysia during the COVID-19 pandemic.

## Materials and methods

This study was conducted during the MCO using a sequential mixed method research design. The mixed method design was used to heighten the “knowledge and validity” [14]. Data collection started simultaneously with the quantitative data collected through an anonymous online survey among 827 respondents [14], and the qualitative data among 19 respondents through in-depth phone and online interviews. The quantitative and qualitative data were analysed separately, and then the results interpreted together. This study was a part of a larger study, SEB-COV, involving four other countries (i.e., Thailand, The United Kingdom, Italy and Slovenia) [15]. The online survey was launched online simultaneously for all countries [15, 16]. Volunteers who participated in the survey were invited to register their interest for the interview in a separate link that took them to a separate website to register their interest and provide their email address and permission to be contacted. Protocols for both the survey and in-depth interviews for the international SEB-COV study were developed collaboratively by the cross-country research teams [14]. The results of the multi-country quantitative and qualitative analyses have been published separately [15, 16]. In this paper, we focus on the Malaysian segment where the qualitative research was extended due to the implementation of mass vaccination. Follow-up interviews were carried out with existing participants to obtain updated data on their vaccination experiences (Appendix 1). The researcher, PKC, had contacted all the respondents for a follow-up interview, consequently four follow up interviews were done. Additionally, one more participant was recruited purposively using snowball sampling, based on their vaccine hesitancy, to provide a broader perspective. These follow-up conversations allowed us to collect current information on vaccination sentiments and experiences in Malaysia.

### Ethics approvals

The cross-country SEB-COV study was approved by the Oxford Tropical Research Ethics Committee (OxTREC, reference no.520 − 20). In Malaysia, approvals were obtained from the Medical Research and Ethics Committee, the Ministry of Health Malaysia (MOH ref: NMRR-20-595-54437 (IIR)), and the Universiti Tunku Abdul Rahman Scientific and Ethical Review Committee (SERC, ref: U/SERC/63/2020).

### Sampling

Adult populations residing in Malaysia were invited to participate in the in-depth interview and anonymous online survey through separate links that was provided in our website. Participants were recruited through email, online platforms and posters using professional and personal networks. Those who volunteered for the in-depth interview provided some demographic information such as their age, profession, and their email addresses so that the researchers could purposively select the sample and contact them for an interview.

### Data collection and analyses

#### Online survey

The survey data were collected between 1 May and 30 June 2020. Recruitment partly relied on a nonprobability sampling technique, in which initial study subjects recruited future study subjects from their acquaintances. Also, with the assistance of a professional polling agency, subjects were contacted via professional networks by emails and listservs. Moreover, the survey was distributed on social media such as Facebook and Twitter.

The analysis utilised a sample that surpassed the planned sample size of 600 per country, resulting in increased precision than desired [17]. The intended 600 survey participants were sufficient to estimate the prevalence of any response with a 95% level of confidence and a precision of 4%, assuming a 50% prevalence rate. The 50% prevalence is the standard assumption for sample size calculations when the true prevalence is not available in literature because this gives the largest required sample size for a binomial distribution for a desired level of precision. Thus, the sample size in the survey in Malaysia, 827, is sufficient to estimate the prevalence of any response with a margin of error of estimated 3% and a confidence level of 95%. Post-stratification weighting was applied to compensate for the non-probability sampling, and data was analysed using Stata 15.0 software. Three stratifying variables obtained from Malaysia’s population census data (i.e., gender, educational level, and age) were employed in computing the weights. As described in a previous study [15], weights were estimated as the ratio between the proportion of each combination of the three variables in Malaysia’s population and the corresponding proportion in the participant sample.

#### In-depth interviews

In-depth interviews were conducted with participants to elucidate the challenges they faced and their coping behaviours during the pandemic and the associated MCO. Participants in the interviews needed access to a phone or a computer with internet. The decision to proceed with online or phone interviews was pragmatic since the MCO was in effect at the time. The interview guide was developed based on the objectives of the study [14, 17]. After serveral online team discussions, the interview questions were fine-tuned. Then, a pilot test was conducted to further develop and improve the interview guide before the actual data collection began. A total of 19 in-depth phone interviews were conducted between 2 and 2020 and 20 December 2021, until data saturation was reached.

We used three ways to recruit the interview participants. Firstly, participants were recruited by providing a link in the online survey website. By clicking the link, a separate webpage that was not linked to their survey responses would open so that the participants could fill in the online form to enable researchers to contact them. Recruitment of participants was also conducted through personal networks and social media by inviting respondents to sign up to volunteer for the interviews using the link to the similar webpage. Lastly, snowball sampling was used to achieve maximum variation and to enable us to contact those who did not have access to social media, computers, or the internet.

The selected participants were contacted, and written informed consent was obtained electronically. Permission was sought to have the interview audio recorded. Each interview conducted in English mixed with local dialects, ranged from 40 to 90 min. After each interview, fieldnotes were taken and the recording transcribed verbatim. The transcripts were coded independently by two researchers (PKC and P-KC). In the initial coding process, researchers read and reread the transcripts. Analysis of the data was done by first employing first cycle coding, then transitioning to second cycle coding [18]. Then researchers assigned a word, or short phrases or extract quotes or in vivo codes from the transcripts to symbolically capture the essence of the data [18]. Next, the codes were arranged in categories, while researchers conducted an iterative process to code, categorise and conduct analytic reflection [18]. Each researcher then reflexively generated the themes, then reviewed, refined and defined the themes [19, 20]. Then the themes were compared and both researchers discussed the slight divergences and collaboratively reviewed and refined the descriptions of the main themes [19, 20].

The trustworthiness of the data was assessed and ensured using Lincoln and Guba’s criteria of credibility, dependability, confirmability, and transferability which have been widely used in various health research [21]. Credibility was achieved by engaging with the participants over an extended period of 20 months to conduct the interviews, and follow-up interviews with a few to seek clarifications when needed. In addition, the investigators are qualified and trained researchers who are familiar with the interview guide after having collectively developed and fine-tuned it [22, 23]. To ensure confirmability, methodological and investigator triangulation techniques were employed. To achieve method triangulation, data from in-depth interviews and survey were integrated to enhance objectivity and verify the findings. Also, investigator triangulation was achieved by having two researchers coding the interview transcripts independently who later met to discuss the variations and nuances in their descriptions of the themes. To ensure findings from this study can be transferred to similar contexts, this study employed purposive sampling to gather information from participants and demographic data – gender, age, level of education, household structure, and household size. Besides transferability, the research methodology is also densely described [21] to ensure dependability.

## Results

### Sociodemographic profile of survey and interview participants

The participants of the online survey were from both the West (Peninsular) and East Malaysia. Table 1 [15] shows the distribution of respondents according to their demographic characteristics including gender, age, level of education, household structure and size. About 42% shared a household with children under the age of 18 years, and nearly a quarter (18.6%) shared a household with a person over the age of 70 years. Meanwhile, the majority of respondents (59.3%) stated that they earned a fixed salary, while only 12.3% stated that they earned “freelance income,” such as daily wages or were self-employed, and 14.4% cited that they did not have a source of income.

**Table 1:** Distribution of respondents by demographic characteristics (N = 827) [14]

Description		n	%
Gender	Male	298	36.0
	Female	525	63.5
	Other	4	0.5
Age (years)	18–34	350	42.3
	35–64	442	53.5
	≥ 65	35	4.2
Level of education	Primary/ Secondary	82	9.9
	Tertiary	745	90.1
Household structure	Living alone	74	9.0
	Living only with partner/spouse	95	11.5
	Living with partner/spouse and children; living as single parent with children	312	37.7
	Living with other relatives/non-relatives/other	346	41.8
Household size	1	68	8.2
	2	121	14.6
	3–5	457	55.3
	≥ 6	181	21.9

The demographic details of the 19 interview participants are shown in Table 2. The level of education, age, gender, job and living situation are described for each participant. Most interview participants had tertiary education and co-resided with others in the same household. The average age of the participants was 47 years, with 8 females and 11 males. Participants were predominantly ethnically Chinese, with some being of Indian and Malay decent.

**Table 2:** Demographic details of the interview participants (N = 19)

Participant	Education	Age	Gender	Ethnicity	Job	Family & Living Situation
P01	Tertiary	37	F	Chinese	Housewife	Lives with spouse and 3 children
P02	Tertiary	42	M	Chinese	Manager	Lives with spouse and 3 children
P03	Tertiary	32	M	Chinese	Government servant	Lives alone at work hostel
P04	Tertiary	44	F	Indian	Graduate student	Lives with 4 children
P05	Tertiary	52	M	Indian	Government servant	Lives with spouse and 3 children
P06	Tertiary	29	F	Chinese	Graduate student	Lives alone at a rented house
P07	Tertiary	48	M	Indian	Site Manager	Lives with spouse and son
P08	Tertiary	64	M	Chinese	Retired	Lives with a family of 6 and a domestic helper
P09	Tertiary	49	F	Malay	Lecturer & Entrepreneur	Lives with a family of 7
P10	Tertiary	32	M	Chinese	Administration officer	Lives with spouse and baby
P11	Secondary	84	M	Indian	Retired	Lives with elderly spouse
P12	Secondary	52	M	Chinese	Businessman	Lives with children and dad who passed away during the MCO
P13	Secondary	70	F	Chinese	Retired journalist	Lives with spouse
P14	Tertiary	63	M	Chinese	Businessman	Lives with spouse
P15	Tertiary	52	F	Chinese	Entrepreneur	Lives with 2 children
P16	Tertiary	19	F	Chinese	University student	Lives with mother and younger brother
P17	Secondary	37	F	Chinese	Staff nurse for 15 years	Lives with spouse
P18	Tertiary	59	M	Indian	Insurance advisor	Lives with spouse and 2 children
P19	Pre-university	34	M	Malay	Civil servant	Lives alone

Four common themes emerged from the analysis of the survey and interview data. Data from both sources are interrelated around the themes of work and business, emotional impact, coping with changeand the COVID-19 vaccine.

### Work and business – “MCO is hard”

The online survey results found that 613 (74.12%) respondents were working before start of the COVID-19 pandemic, whereas 214 (25.88%) were not. Among those working before COVID-19, Table 3 shows the types of income and impact of COVID-19 on their income.

**Table 3:** The economic impacts by type of income (N = 613) [14]

If you were working before COVID-19, has COVID-19 created any inconvenience for you?	Economic impacts and economic concerns type of income (*f*)						Total (*f*) (n = 613)	
	Fixed salary (n = 475)		Contract and freelance (n = 125)		Other/no income (n = 13)			
	*f*	%	*f*	%	*f*	%	*f*	%
Loss of earnings	69	14.5	79	63.2	7	53.8	155	25.3
Loss of job	18	3.8	24	19.2	2	15.4	44	7.2
Reduction of working hours	163	34.3	60	48.0	5	38.5	228	37.2
Closure of workplace	214	45.1	67	53.6	8	61.5	289	47.1

Table 3 [15] shows that the highest proportion of respondents in each category of income reported being inconvenienced by the closure of the workplace, followed by the reduction of working hours. By contrast, data gathered from interviews suggested that the closure of the workplace caused some participants to have to work more hours:

*“…in the past few weeks right…we don’t need to go to the office physically, and everything works from home… to be honest, during this period right, our workload was actually more. Not less, but more because we have to prepare even more reports for the management to make decisions.” (P10)*

Meanwhile, because people could not operate at their workplaces, they had to adapt by working from home for example by conducting meetings and discussions online or setting up a home office. Some participants reported coping well with the adjustment to home working:

*“So, we have a stable set up and the house is able to cater to everyone. We have living space and we have workspace because all of us are working from home and so we can cope quite comfortably.” (P08)*

*“So because of all these [pandemic and lockdowns] I can already make plans, you know. Sometimes I can have online meetings with my customers, make life easier for me so sometimes things will be easier for me also.” (P18)*

However, others, found this more challenging given the different capacities to explore other means of reaching their clients and suppliers.

*“Meeting clients and now you have to use phone calls. When we talk to suppliers, we can’t meet face-to-face, we have to call them and we have to [arrange for] delivery mostly through Grab or this LalaMove [delivery services]. A lot of things need to be arranged now. I would say, there’s a big change and a lot of challenges because there is a lot of restrictions so… I have to plan properly… we are not allowed to work in the office, so there are a lot of things that we need to postpone and cancel.” (P02)*

Participants who were providing essential services did not experience closure of the workplace. For example, P08, a retired healthcare worker, reported occasionally needing to go to the hospital to carry out voluntary work “helping anybody who needs some medical concierging” during the pandemic. Another respondent who provided essential services said that they feared having to go to work because of resulting risk of contracting COVID-19:

*“Despite being afraid of the daily cases reported around my residential area... I still have to go to the office …I have to take more precaution and sometimes I have to give some advice to my subordinates …to [take] some precaution” (P03)*

The survey also found that those who depended on contract and freelance work and those who had other income or no income were equally affected by the loss of earnings (see Table 3). Narratives gathered during the interviews supports this finding coping very well with the adjustment to home working:

*“Let’s say they’re going to close up everything, then what about the economy? …for those with a fixed salary, they have no reason to worry since their salary is uninterrupted. But how about, those like us, depending on contracts or daily jobs to get our income. In fact, we’re badly affected. There are two groups of people, the government servants, they are very happy because they are getting the salary. And then I think the private sector they are a bit stressful, like some of my friends…their companies actually told them that they have to agree to the pay cut, if not [in] three months, they [will have to] close up the business” (P04)*

### Emotional impact – “quite scared to go out”

Many participants reported that the pandemic and lockdown had had an emotional impact on them. Respondents feared getting exposed to or contracting COVID-19. Additionally, not adhering to public health measures (PHMs) appeared to inflict fear among respondents. The MCO banned social gatherings, disallowing people to have large gatherings for any reason to prevent clusters of infection. One respondent said he was “quite scared to go out” (P11) as this could risk him getting exposed to the virus.

*“Like, when we go to Econsave and Tesco [supermarkets] and we see so many people there, we just go to Speedmart [convenience store]and buy whatever we need. We’re scared to go in.” (P09)*

*“I am watching TV every day. About the lockdown, see what happens. And then see one day, 600 people get buried. Knowing this and safety is above all else. And then the worst part is, there is this thing about the people, a group can be infected but there are no symptoms. They are infected but they don’t cough…they don’t have symptoms… but they go around spreading the virus.” (P13)*

Due to the MCO, respondents expressed feeling frustrated, tied, and a sense of loss because of the constraints experienced due to the public health measures and restrictions imposed.

*“Before MCO started... I was quite active, I used to go out a lot. I will go to the library, I will go to the temple, and then I will visit my friends. I’ll go to town and meet my friends, and chit-chat in the coffee shop. But now, I’m tied you know...” (P11)*

*“…able to see that the petrol price is dropping RM 1.25 but not able to pump the petrol. So, it’s like… I want to go out but I can’t go out.” (P01)*

*“I think the negative thing is everything is restricted, not allowed to go out, having dinner outside, you know, going travels, yeah. You know, jogging in the evening, going hiking, everything is restricted. So your daily outdoor activities, because ah, I loved to go outdoors, hiking, jogging and things and all. So that is the main thing that affects ah, my personal life.” (P17)*

The frustration that was felt by the respondents was also said to be due to the limited activities which they could perform within the confines of their homes. P12 expressed that therefore, the only activities that he could settle for were eating and sleeping at home.

*“Oh. You eat, sleep and eat.]. We had to. We had nothing to do. Correct. We had nothing to do. We cannot go out. We cannot meet people. Yeah. You have to eat sleep, sleep and eat. Yeah. Okay. Get on with it.” (P12)*

Apart from feeling frustrated with their daily routines, some respondents expressed feeling a sense of loss and deprivation. Participants felt that they could not do the things that they wanted or used to do and a sense of loss and missing out on the things they could not do due to the pandemic. One participant said the opportunity to participate in meaningful, enjoyable and important activities and routines that were a part of his life before the pandemic had been “taken away” (P09), further explaining:

*“Previously, if it’s anyone’s birthday, we would like to eat outside, but now we can’t do that. So, we feel like now that’s been taken away, more limited choices.” (P09)*

The sense of loss also includes missing out on important events and milestones in life such as celebrations and events. As gatherings and inter-state crossings were not allowed for non-immediate family members, his relatives and friends could not attend the wake to pay their last respects. He felt a sense of loss as such events cannot be postponed. Similarly, another participant experienced missing out on milestones, saying:

*“Oh, sure, a lot of difference. Presently, we just send greetings on phones to a few friends. Not like before, we can go to the temple, meet friends personally, shake hands and wish them [a happy new year], pray at the temple itself... special prayers. Even my own grandchildren’s birthday I couldn’t attend.” (P11)*

### Coping with change – “I have online lunch with my friends”

Most participants reported a lifestyle change, which included alterations in the way that they lived, worked, and socialised with the rules of the MCO, such as travel restrictions, school closure, limited business operations, and closure of places of worship and recreation. Thus, people in Malaysia had to stay at and operate from home. Although people were confined to their homes, our survey found that more than half of the respondents posited they could cope without meeting their friends and family in person, without going out in public and except for essential needs or work for more than four weeks (Table 4) [15].

**Table 4:** Maximum number of days that people could cope during the MCO (N = 827) [14]

Description	n	%
**Time to cope without meeting family or friends in person**		
1 to 14 days	201	24.3
> 14 to 28 days	110	13.3
29 days+	516	62.4
**Time to cope without going out in public**		
1 to 14 days	270	32.7
> 14 to 28 days	114	13.8
29 days+	443	53.6
**Time to cope without going out except only for essential needs/work**		
1 to 14 days	268	32.4
> 14 to 28 days	98	11.9
29 days+	461	55.7

Most respondents coped by connecting with others, engaging in their hobbies or learning new skills, finding alternative ways for things they enjoy, self-care and watching movies or series (Table 5) [15].

**Table 5:** The frequency of coping strategies (N = 827) [14]

Description	*F*	% (n = 827)
Connecting with others	808	97.7
Engage in hobbies or learn new skills	700	84.6
Finding alternative ways for things I enjoy doing	729	88.2
Self-care	729	88.2
Watching movies or series	757	91.5
Total *f*	3723	97.7

Since most places that allowed social gatherings (i.e., places of worship, restaurants and recreation and sports facilities) were closed during the MCO, interview participants had to find alternative ways to continue their activities.

*“I play badminton a lot so like every week at least like three or four nights outside until like 10 o’clock. But now, we’re not allowed to go out after eight, so we need to stay at home obviously not allowed to play badminton as well, so I have to make sure I keep my fitness and also do more exercises [at home] to keep myself healthy.” (P02)*

*“I used to go out, meet friends all these things. Nowadays, I just stay at home. I just spend my time just reading newspapers, watch television. Just relax.” (P11)*

Moreover, being confined to their homes, almost all respondents (96.74%) stated that the use of the internet and online social networks were important or very important to them. For example, a leader of a temple explained that since the temple had to be closed, many people resorted to praying at the fence of the building. As a result, live streaming was later organised to enable worshippers to participate in temple activities online:

*“Once in a while, I go to the temple in the evening, and I tell them they cannot be gathering here, so we started doing some live streaming.” (P05)*

In contrast to the habit of eating out before the pandemic, several participants expressed they prepare meals at home more often during the MCO given the travel restrictions and government advice to stay home. Doing so would also minimise or avoid the risk of exposure to COVID-19 although it deprived them of their favourite meals from restaurants.

*“I have a new routine. Of course, I wake up a bit later than usual, and then… I cook breakfast every morning, sometimes… I still can go to Family Mart or 7-Eleven [convenient stores] to grab something for the children if I’m too tired, but now I am totally unable to do that. So, it’s all about cooking, cooking and cooking.” (P01)*

*“Because previously I don’t cook every meal. I cook maybe like, two, three, days in a week. This time, I cook every day. Literally every day and every meal.” (P06)*

*“I think the difficult decision was in terms of like, initially we were like ordering food, using GrabFood to minimize the contact, because I don’t expect my wife to cook every day. I mean I cook as well, so we don’t expect to cook every day. Sometimes, you want to eat something from outside, but now we can no longer buy food like how we did before the pandemic, and we need to minimize contact with outsiders.” (P07)*

Cooking meals at home was also perceived by some to have brought about positive effects, as one respondent explained that eating homecooked meals means eating healthier.

*“I’m getting healthy food now. My wife tends to cook daily, until and when I tell her, today you take a break, then we’ll buy food.” (P05)*

As evident from the quotes above and other participant interviews, women appeared to bear the majority of the extra work involved in cooking and preparing meals during lockdown.

People also reported using online and social media to maintain their social relationships by connecting with others.

*“Ah, you see, because I’m at this age, all my ex-colleagues are all same age, about the same age, so we go WhatsApp. WhatsApp becomes very useful.” (P13)*

*“During MCO, I don’t go to campus... so I have online lunch with my friends.” (P06)*

### The COVID-19 vaccine – “I am forced to take the vaccine”

Participants demonstrated mixed feelings and expressed different perspectives regarding the COVID-19 vaccine. Some were in support of taking the vaccine while others were vaccine-hesitant. For instance, the national uptake of the vaccine as of July 2021 over the duration of the in-depth interviews was 54% but at least 80% of adults have received a COVID-19 vaccine as of February 2022.

*“They told us it is voluntary, in the form they also say it is voluntary, but in reality, they force us. I am forced to take the vaccine… I will try to avoid it for as long as I can. But if they threaten to suspend me from my job, I will still have to take it. I have no choice.” (P19)*

Some respondents felt that the COVID-19 vaccine does more harm than good.

*“Vaccine depends on the body of the people. There are many side effects. There are no advantages only disadvantages such as death, damages, and disabilities. One of my colleagues without any history of heart disease got a heart attack after getting the vaccine, another one got gastric issues. Is this a coincidence?” (P19)*

*“It is not compulsory to take. If possible, I don’t want it. I am so scared. I had allergies, I hear people say for boosters, the effects will be worse. I heard from people who had it.” (P09)*

Two respondents expressed that taking the COVID-19 vaccine is risky as they believed it had not passed sufficient clinical trials.

*“It’s not going to be a cut and dried um, vaccine story, because we have seen vaccines that have not been successful, notably in dengue virus, and therefore the same thing can happen to this virus, this particular virus. In the case of the Influenza virus, there are many vaccines available, but each year, different viruses can crop up, can mutate, which means the previous vaccines may not be effective. So, the vaccine is not going to be the ready answer.” (P08)*

*“Vaccine is still in the trial stage… if something goes wrong, they don’t give us compensation. We suffer the consequences ourselves…The only thing we can do is protect ourselves. For those who took the vaccine voluntarily. They did not do enough research, or they did not get enough information. Maybe the information came late. It is their luck if nothing happens, then they are lucky…The vaccine may cause more harm than good.” (P19)*

Other respondents were less apprehensive and welcomed the vaccine as it offered some protection from the virus.

*“Now I feel that I already have the 2 doses, so it is enough. And I will follow the SOPs so it is quite safe.” (P09)*

*“When the government announced that the vaccine was available to everyone, I stayed up until midnight to wait for the website to open so that I could book an appointment. All my friends were also eagerly waiting for the vaccine.” (P06)*

## Discussion

This study presents in-depth information on the lived experiences and views of residents in Malaysia on the COVID-19 pandemic, the impact of public health measures implemented during the MCO, and perceptions toward the COVID-19 vaccine. Participants in this study held diverse views about the government’s response, especially the implementation of various phases of movement restrictions across the country. However, most participants posited experiencing significant challenges during the period, ranging from financial to social constraints, fears, and loneliness. As a result, they engaged in diverse coping strategies to address the challenges emanating directly from the pandemic and indirectly from the measures enacted by the government. These coping strategies comprised practical and socio-cultural ways of tackling the issues.

### Challenges

Most participants in the survey and interview sessions reported experiencing significant financial, social and emotional challenges during the pandemic. Specifically, the drastic loss of income and reduction of working hours indicates that COVID-19 affected both people on fixed and flexible salaries. For instance, the informal sector workers and tourism account for more than half of the workforce population in Malaysia, contributing about 15% of the GDP before the pandemic [13]. Without economic recovery, the Malaysian Institute of Economic Research projected that the country’s economy would fall from 4% to -2.9% in 2020, resulting in up to 2.4 million job losses, of which 67% would be among low-skilled workers [10]. According to Bank Negara Malaysia (National Bank of Malaysia), GDP growth in 2020 was expected to range between − 2.0% and 0.5%, owing to direct output losses caused by the COVID-19 pandemic, the enactment of the MCO, and interruption in both domestic and international resource supply [10]. Hence, it is unsurprising that most participants in this study reported experiencing significant economic losses during the period in which government measures were introduced to control the pandemic. Aligning with the government’s projection, both low-skilled and educated individuals experienced various degrees of financial challenges as reflected by the results of this study. These findings are consistent with reports from studies conducted in Thailand and Vietnam in which financial drawback was the predominant concern raised by residents during the first and second waves of COVID-19 [22–24].

While financial interventions and packages have had a cushioning effect on reducing the financial impact of COVID-19 in many countries, little to no impact of such interventions was reported in others [25]. For instance, due to the lack of governmental financial support in Vietnam, Thailand, and Laos, vendors experienced a sharp decline in income and source of livelihood following the public health restrictions imposed by the government [24, 25]. Meanwhile, the Malaysian government announced stimulus packages totalling approximately 60 billion United States Dollars (USD), or roughly 17% of the counry’s GDP following the first wave of the pandemic and MCO to fund loan deferments, one-time income support, and direct fiscal infusion into the economy [1]. Although participants in studies conducted elsewhere highlighted similar financial challenges as a result of a lack of government support [24], the case of Malaysia is unique in that stimulus packages were provided for low-income and vulnerable populations. Nonetheless, participants of this study still reported financial challenges, indicating the momentous impact of income losses during the MCO.

Furthermore, findings from this study suggest the need for the government to expand their financial and social support programmes to meet the needs of vulnerable and often neglected populations who are likely to disproportionately suffer the adverse impacts of COVID-19 public health policies. Examples include informal workers, freelance workers, transnational migrants, older and younger people, those residing in large households, and individuals living in informal settlements [15, 23, 26]. For instance, the present study revealed that approximately 15.0% of the surveyed respondents were freelance workers while 13.0% were completely unemployed or working in the informal sector. Failure to capture such vulnerable groups in government support programmes is likely to have ripple effects on a larger population in the country. A comprehensive public health approach, which considers the lived reality of marginalised populations is required while addressing social predictors of health, health system preparedness, and social protection mechanisms [27].

The second theme in this study pertains to the emotional impact of the pandemic as most participants remained indoors and were afraid to go out except when purchasing necessary goods or during emergencies. Qualitative findings highlight the emotional impact of COVID-19 among respondents, which stemmed mainly from the fears of getting exposed or contracting the virus. These findings coincide with the reports from similar research conducted in Thailand, Slovenia, and Vietnam [15, 24] in which fears and anxiety about COVID-19 infection contributed significantly to the emotional impact of the pandemic. In our study, not adhering to public health measures further inflicted fear among participants, leading to isolation and loneliness as reported in other studies [24, 26]. Although these emotional impacts have been associated with severe consequences such as post-traumatic stress disorder, suicidal ideation and suicide in other studies [28], the most severe implications expressed by participants in our study were feeling frustrated, tied, and a sense of loss mainly due to the constraints experienced because of restrictions imposed. For instance, the ban on social gatherings limited several activities and hobbies that kept people engaged before the pandemic. Nevertheless, most participants reported adhering to the government rules guiding the MCO due to fear of contracting the virus.

While sharing their lived experiences of isolation and loneliness as pertinent challenges, we observed that these events were strongly linked to fears of infecting loved ones or contracting the virus themselves. These results corroborate the studies conducted in Thailand, where limited social contact arising from an interruption to social interactions was considered the most challenging effect of COVID-19 public health measures [14, 25, 27]. Malaysians have a strong affection for close family and community ties which is evident in frequent social interactions organised by various races in the country. Important cultural events were immensely interrupted by social distancing guidelines during the first and second phases of the pandemic [22, 23]. Overall, our findings highlight the psychological implications of COVID-19 measures as reported by local [23] and international scholars [24, 28].

### Coping strategies

The coping strategies employed by participants to address the negative effects of public health measures implemented by the government were mainly two-fold; practical and socio-cultural lifestyle changes. Practically, participants engaged in social distancing and physical guidelines recommended by health agencies to prevent exposure. They also took active steps to care for their emotional well-being, and mental health, and reduce the economic implications of the public health measures. Examples included connecting with others via the Internet, engaging in hobbies or learning new skills, self-care, finding alternative ways for things they enjoy, and watching movies or series. The pandemic was a shock to the world, but it also catalysed novel and creative approaches which might strengthen individual and community resilience [12]. Recent research conducted in Malaysia found that the pandemic did not only create adverse impacts but also offered new public health strategies to help people cope with their mental health needs in a more optimistic manner [29–31]. The MCO changed people’s lifestyles while adapting to new norms to curb the pandemic [32, 33]. However, most coping activities were not substantiated in this study except the increased Internet usage for communication, work purposes, and in some cases, religious activities.

The most profound usage of the Internet was relayed by a participant following the switch from normal visits to Indian temples to online sessions, thereby representing socio-culturally embedded practices to cope with COVID-19. Our results echo other findings on the relationship between social support and religious coping in the context of COVID-19 [15, 29]. Likewise, a qualitative study conducted in India to assess the coping strategies and mental health among disadvantaged people during the pandemic revealed the crucial role of religion and devotional practices in supporting people address the incessant challenges [30]. A study in Malaysia has also highlighted the coping strategies adopted by individuals from other religious faith such as Islam and Christianity [31, 32, 34], but such findings were not gleaned from the present study. This discrepancy might be due to the fact the former studies focused on religious coping strategies which enabled participants to share their lived experiences on such matters.

### COVID-19 vaccine

Understanding COVID-19 vaccination intentions and perspectives are pertinent to achieve population-level immunity and putting an end to the pandemic [35]. Ever since the first COVID-19 vaccine was introduced, it has been received with some scepticism due to perceptions about rapid vaccine development, the use of novel technologies, and prompt registration in less than a year [15, 36–38]. Similar findings were reported in studies conducted in Jordan and Thailand [33, 36] as participants exhibited safety concerns about the potential side effects of COVID-19 vaccines.

A safety trial was organised by the Malaysian government in order to address the concerns associated with the COVID-19 vaccine and its uptake among residents [39]. Notwithstanding, several participants in this study were vaccine-hesitant, which aligns with the findings from research conducted in the United States [38] during the initial roll-out phase of COVID-19 vaccines as participants still raised concerns about the side effects and opined to wait and see if the vaccine is safe.

Since we did not specifically investigate the predictors of vaccine acceptance, motivation, and barriers among the participants, the underlying factors for vaccine acceptance or hesitancy among participants of this study are not well understood. Nevertheless, some of the reports in other related studies might provide a clue. Moorty et al. [37] concluded that ‘most Malaysians remain optimistic and support the government’s immunization initiatives. Recent studies revealed that mistrust of the government’s management of the national vaccination programme and lack of transparency in disseminating information regarding the efficacy of the vaccines and their contraindications were consistent factors associated with vaccine hesitancy [35, 40–42]. These factors might equally contribute to participants’ views in the current study as safety concerns are often linked to uncertainties surrounding the vaccine efficacy and distrust of the information disseminated by government agencies. As a result, participants resolved to take the vaccine shots to abide by the policies enacted rather than a form of a disease prevention programme. Meanwhile, educational status has not been identified as a factor influencing COVID-19 vaccine hesitancy or uptake in Malaysia [43]. Our results also appear to support such findings as most participants in the survey and interview were highly educated; yet diverse views were expressed regarding the efficacy and readiness to be vaccinated.

### Strengths and limitations

Our study is one of the few studies that examined the lived experiences, coping strategies and perspectives of people in Malaysia regarding the impact of COVID-19 and its associated public health measures. Additional strengths in this study include the use of a mixed method approach and the application of weighting strategies of the survey samples during the analysis. These procedures enabled us to gather robust data from the participants, which is a step forward relative to previous local studies that utilised either a quantitative or qualitative method. For the survey, participants’ characteristics such as socio-economic status, educational levels, whether or not they have/had COVID-19 and other health-related information were not gathered. This was a strength and also a limitation. We hoped that participants were more honest in their answers and were more willing to participate because minimal personal information was collected. Indeed, our sample size exceeded our target sample size of 600. The limitations of this strategy are well-acknowledged, for example we could not compare the responses of who have experienced COVID with those who have not.

Other limitations are also noted. The self-reported nature of the survey may also introduce response bias. Face-to-face data collection could not be conducted due to COVID-19-related restrictions, thus individuals who were either illiterate or lacked access to online technology were not considered in this study. Hence, the present findings may align towards economically advantaged and more educated persons in Malaysia. Despite using weighting strategies during the data analysis, results from this study are not generalisable to Malaysia’s population since the study did not aim to recruit a nationally representative sample. Other limitations include response bias emanating from recall, self-reporting nature, and using cross-sectional study designs, reflecting that our data elucidate the prevalence of specific events and opinions rather than any form of causality. As for the qualitative part of the study, we acknowledge that most of our participants have tertiary education and of Chinese ethnicity. We were not able to conduct in-person interviews due to the MCO hence those we could only interview people who had mobile phones or computers with internet access.

## Conclusion

This study provides insights into the experiences, coping strategies and perspectives of people in Malaysia living through the first-ever MCO during the COVID-19 pandemic. Such insights into lived experiences of COVID-19-related public health measures are important for successfully planning and implementing responses to future pandemics. Participants in this study shared different perceptions and experiences on the measures implemented, indicating that COVID-19 measures had unequal impacts on different social groups. These findings are important in assisting policymakers and healthcare administrators better understand the impact of these public health measures. Our data contributes to the body of evidence needed to inform context-specific policy decisions in future COVID-19 waves and future pandemics.

The extent to which various sectoral groups accept and adhere to stringent public health measures is crucial for mitigating the spread of COVID-19 and optimising societal outcomes during the present COVID-19 outbreak. With the introduction of the national immunisation programme as recommended by the World Health Organisation (WHO), the issues of vaccine hesitancy need to be elucidated by identifying factors contributing to these perceptions among the population. Building people’s trust in the government’s management of the national vaccination programme and ensuring transparent dissemination of information on the vaccine efficacy and its potential side effects may assist in addressing some of these issues.

Our study demonstrates unequivocally that public health measures impacted various social groups in Malaysia during the COVID-19 pandemic. Furthermore, the results indicate which groups are most impacted by the pandemic in its early stages, necessitating protection from further health and social inequities. Thus, it is critical to consider appropriate health and socio-economic protection schemes such as financial stimulus packages and mental health services for targeted populations including informal sector workers and young people.

## Electronic supplementary material

Below is the link to the electronic supplementary material.

Supplementary Material 1

## Data Availability

Data are available upon reasonable request to the Mahidol Oxford Tropical Medicine Data Access Committee (email: datasharing@tropmedres.ac). Data is not available from the corresponding author. All authors recognise the value of sharing individual level data. We aim to ensure that data generated from all our research are collected, curated, managed and shared in a way that maximises their benefit.
