# Supplementary material for: Experiences, coping strategies and perspectives of people in Malaysia during the COVID-19 pandemic
Source: BMC Public Health. 2023 Jun 6;23:1085. doi: 10.1186/s12889-023-15892-5 (PMC10242592; doi:10.1186/s12889-023-15892-5)
Supplement: Supplementary file 1 — Supplementary Material 1 [file 12889_2023_15892_MOESM1_ESM.docx]

**Appendix 1**

**Topic Guide for Follow-up Interview**

1. How have you been since I last spoke to you?

2. How do you feel today?

3. Can you tell me what has changed in your life since the last I spoke to you?

4. What is your opinion on the Covid-19 vaccine?
